# Lack of MRE11-RAD50-NBS1 (MRN) complex detection occurs frequently in low-grade epithelial ovarian cancer

**DOI:** 10.1186/s12885-016-3026-2

**Published:** 2017-01-10

**Authors:** Simone Brandt, Eleftherios P. Samartzis, Anne-Katrin Zimmermann, Daniel Fink, Holger Moch, Aurelia Noske, Konstantin J. Dedes

**Affiliations:** 1 Institute of Surgical Pathology, University Hospital Zurich, Zurich, Switzerland; 2 Department of Gynecology, University Hospital Zurich, CH- 8091 Zurich, Switzerland; 3 Kempf & Pfaltz, Histologische Diagnostik ag, Zurich, Switzerland

**Keywords:** Ovarian cancer, MRN complex, PARP inhibitor

## Abstract

**Background:**

BRCA1/2-deficient ovarian carcinomas are recognized as target for Poly (ADP-ribose) polymerase (PARP) inhibitors. BRCA1 and BRCA2 proteins are involved in homologous recombination repair of double-strand DNA breaks. The relevance of other homologous recombination repair proteins, e.g. MRE11, RAD50, NBS1 (MRN complex) in ovarian carcinomas is unclear. The objective of this study was to investigate the prevalence of lack of MRE11, RAD50, NBS1 protein detection in epithelial ovarian cancer (EOC).

**Methods:**

A tissue microarray (TMA) with 134 EOC was immunohistochemically evaluated for MRE11, RAD50 and NBS1. Data was analysed for associations with clinicopathological parameters, histological subtype, patient overall survival and mismatch repair (MMR) protein status. Sensitivity towards the PARP inhibitor BMN673 was tested in two ovarian cancer cell lines (TOV-21 and OVTOKO) using colony formation assays.

**Results:**

Lack of MRN complex protein detection was seen in 41% (55/134) of EOC and was more frequent in low-grade (57.6%; 19/33) than in high-grade EOC (18.8%; 36/101; *n =* 134; *p =* 0.04). There was an association with the ovarian carcinoma subtype (60.3%; 35/58 lack of detection in type I versus 26.3%; 20/76 in type II; *n =* 134; *p <* 0.001) as well as undetectable DNA mismatch repair proteins MLH1 and MSH2 (89.3%; 25/28; *n =* 131; *p <* 0.001). MRE11 knockdown led to moderately increased sensitivity towards the PARP inhibitor BMN673 in one ovarian carcinoma cell line in vitro.

**Conclusions:**

Frequent lack of MRE11, RAD50, NBS1 protein detection in type I human ovarian carcinomas is observed in EOC and our data suggests further investigation regarding sensitivity to PARP-inhibition in tumours lacking MRE11 expression.

## Background

Epithelial ovarian cancer (EOC) represents about 30% of gynaecological cancers and is the leading cause of gynaecological cancer death in the Western world [1]. Patients with EOC often present in an advanced disease stage [2]. The current treatment strategy is surgery followed by platinum-based chemotherapy [3]. Due to resistance to chemotherapy and recurrence of the disease, long term survival of ovarian carcinoma patients remains poor.

Histologically, EOC is a heterogeneous group including low- and high-grade serous cancer, mucinous, endometrioid and clear cell cancer. Germline *BRCA1* and *BRCA2* mutations account for about 10–15% of ovarian cancers and are mainly found in high-grade serous and endometrioid ovarian carcinomas [4, 5]. BRCA1 and BRCA2 are critical proteins in the process of homologous recombination repair (HR) of double-strand DNA breaks. In addition to BRCA1 and BRCA2, many other proteins are involved in the HR repair process of double-strand DNA breaks and are implicated in hereditary breast and ovarian cancer susceptibility. Such genes include *ATM, CHEK2, BARD1, BRIP1, MRE11, RAD50, NBS1, RAD51C, RAD51D and PALB2* [6]. In the initial stages of HR, a double-strand DNA break is recognized by ATM and ATR, kinases that phosphorylate downstream targets including p53 and BRCA1. BRCA1 acts as a scaffold that organizes the remaining proteins to the site of repair. In a second phase of HR repair of double-strand DNA breaks, the MRN complex, which consists of MRE11, RAD50 and NBS1, resects the DNA to form 3″ overhangs. This is followed by loading of RAD51 onto RPA-coated DNA under the influence of BRCA2 [7–9].

The MRN complex can be inactivated or impaired by mutations or epigenetic silencing occurring in one of its three components. Homozygous *MRE11* and *NBS1* germline mutations that cause a lethal phenotype in mice are rarely encountered in humans and lead to an Ataxia telangiectasia-like disorder (ATLD) and Nijmegen breakage syndrome (NBS), respectively. Heterozygous germline mutations of MRN complex genes may be associated with breast and ovarian cancer susceptibility [10–13].

In recent years, HR repair of double-strand DNA breaks has become a target for cancer therapy because BRCA1/2-deficient cancers are recognized as a target for a class of drugs known as PARP (poly (ADP-ribose) polymerase) inhibitors [14, 15]. PARP inhibitors work through direct blocking of PARP enzymatic activity. PARP represents a family of enzymes involved in base excision repair (BER), a key pathway in the repair of single-strand DNA breaks. Three excision repair pathways exist to repair single-stranded DNA damage: Nucleotide excision repair (NER), base excision repair (BER) and DNA mismatch repair (MMR). Loss of DNA MMR proteins is seen in hereditary non-polyposis colorectal cancer (HNPCC), but also in ovarian carcinomas [7, 8, 16].

In the situation of PARP inhibition, single-strand DNA breaks are converted into double-strand DNA breaks through collapse of the replication fork. In BRCA-deficient tumors, homologous recombination repair is not functional. Therefore, the deficiency of both, HR repair of double-strand DNA breaks and single-strand DNA damage repair due to PARP inhibition leads to death of tumor cells. The term synthetic lethality means that deficiency of PARP or BRCA alone has no impact, but a deficiency in both leads to a lethal effect in tumor cells because the tumor cells are directed towards error-prone repair and consecutive cell death [6].

In the last years, PARP inhibitors have shown promising results among *BRCA1/2* mutation carriers, among them several completed trials for PARP inhibition in *BRCA1* and *BRCA2*-mutated patients with EOC [14, 17, 18]. A completed study in patients with relapsed high-grade serous ovarian cancer showed improved progression free survival among patients with platinum-sensitive relapsed tumors, but no overall survival improvement [19, 20].

In sporadic tumors, genes in DNA repair may also be altered due to somatic mutations or epigenetic alterations. In high-grade serous EOC, up to 50% harbor disruption of HR by mutations or epigenetic silencing of *BRCA1/2, RAD51* and others [21] and up to 29% of EOC harbor defects of MMR [22, 23]. The Cancer Genome Atlas (TCGA) suggests that deficiency of either BRCA1 or BRCA2 occurs through somatic mutation (3% *BRCA1* or *BRCA2*) or through epigenetic silencing of *BRCA1* (11%) in sporadic EOC. Other genetic changes affecting HR repair include amplification of *EMSY* (8%), deletion/mutation of *PTEN* (7%), hypermethylation of *RAD51C* (3%), mutation of *ATM* or *ATR* (2%) or mutation of other HR genes (5%). These tumors have the phenotype of “BRCAness” and are predicted to act like BRCA-deficient tumors despite wild-type germline BRCA1 and BRCA2 genes. Such BRCA-deficient ovarian cancers show improved survival, due to a better response to platinum chemotherapy [6].

In vitro experiments have demonstrated that deficiency in HR by mutations in *MRE11*-*RAD50-NBS1* (MRN) complex may sensitize cancer cells to treatment with PARP inhibitors [24–26] and might therefore serve as a predictive biomarker of PARP inhibitor therapy. There is some growing body of evidence that patients with other mutations than *BRCA1/2* may also benefit from PARP inhibitors [15, 27–29].

So far, the expression pattern of the MRN complex in gynaecological carcinomas is not well elucidated. Due to the key role of the MRN complex in HR of double-strand DNA breaks**,** the aim of our study was to evaluate the prevalence of absent protein staining of the MRN complex (MRE11, RAD50 and NBS1) in EOC.

## Methods

### Tissue microarray

Tissue microarray (TMA) with formalin-fixed and paraffin embedded ovarian carcinomas was previously constructed [30]. The study was approved by the local scientific ethics committee (KEK-ZH-Nr: StV 27–2009) and the need for individual consent has been waived by the ethics committee. 144 cancer samples of the archive of the Institute of Surgical Pathology, University Hospital Zurich (Switzerland) were included in this study. Clinical and pathological characteristics were taken from the clinical databases and pathology records. Routine hematoxylin and eosin sections were performed for histopathological evaluation. The stage of tumors was assessed according to the International Federation of Gynaecology and Obstetrics (FIGO) and TNM staging system. Histological subtype and tumor grade was defined according to the WHO classification 2014 [31]. Low-grade serous carcinomas were excluded due to low sample number. The histological grade was classified as low-grade (including well to moderately differentiated endometrioid and mucinous carcinomas) and high-grade (combining high-grade serous, clear cell and poorly differentiated endometrioid cancer). In addition, we classified all EOC according to the Kurman model with two types of progression pathways as type I ovarian carcinomas (low-grade serous cancer, mucinous, endometrioid and clear cell cancer) and type II ovarian carcinomas (especially high-grade serous cancer) [32]. Follow-up data is known from all patients. The mean follow-up time was 56.6 months (range 0.13–201.2 months). Data on adjuvant chemotherapy were known for all patients. Adjuvant chemotherapy was administered in 102 women and was mainly platinum based (68%). The remaining 42 patients (29.2%) did not receive any chemotherapy after surgery. Resistance to chemotherapy is defined as disease progression or recurrence within 6 months after end of therapy / within a 6 month therapy-free interval. Sensitive status was defined as a therapy-free interval of at least 6 months without evidence of tumor progression or recurrence. In our cohort, 50 (34.7%) cases were classified as sensitive (relapse >6 months) and 32 (22.2%) as resistant (relapse <6 months). In 20 (13.9%) cases the response status could not be determined.

Baseline characteristics of patients with ovarian cancer are summarized in Table 1.

**Table 1:** Clinicopathological characteristics of ovarian carcinomas (*n =* 144)

Variable	*n* (%)
Age at diagnosis (*n =* 144)	
≤ 60 years	67 (46.5)
> 60 years	77 (53.5)
FIGO stage (*n =* 137)	
Early (I & II)	44 (32.1)
Late (III & IV)	93 (67.9)
Histological subtype (*n =* 144)	
High-grade serous	73 (50.7)
Mucinous	15 (10.4)
Clear cell	26 (18.1)
Endometrioid	30 (20.8)
Grade (*n =* 144)	
Low-grade	36 (25)
High-grade	108 (75)
Intraoperative residual tumor (*n =* 98)	
< 1 cm	27 (27.6)
> 1 cm	71 (72.4)

### Immunohistochemistry

After antigen retrieval, the slides were incubated with the following antibodies: MRE11 (clone 31H4, cell signalling, no.4847, 1:500), RAD50 (13B3/2C6, Abcam limited, no. ab89, 1:500), NBS1-p95 (cell signalling, no. 3002, 1:50). After incubation for 1 hour at room temperature, the staining of MRE11, RAD50 and NBS1 was further conducted with the Ventana Benchmark automated system (Ventana Medical Systems, USA) using Ventana reagents as well as the UltraMap™ DAB detection kit as described previously [24]. Analysis of all stainings was independently performed by two pathologists (SB, AN). Nuclear immunoreactivity of MRE11, RAD50 and NBS1 was scored as: negative (0), weak (1), moderate (2) and strong (3). Stromal cells showing nuclear staining were used as a positive control.

The antibodies against the mismatch repair proteins MLH1 (G168-15, PharMingen, Becton Dickinson, 1:100) and MSH2 (25D12, Novocastra Lab. Ltd, 1:100) were incubated for 30 min and the staining procedure was carried out with the automated Leica BOND system using the Bond Polymer Refine Detection Kit (Leica Biosystems) as described previously [24]. The protein detection of the mismatch repair genes was considered positive when nuclear staining was evident. Stromal and inflammatory cells showing nuclear staining served as a positive control.

### Cancer cell lines and growth conditions

The ovarian clear cell cancer (OCCC) cell lines TOV-21 and OVTOKO were grown in RPMI supplemented with 10% (v/v) fetal bovine serum and 1% (v/v) antibiotic-antimycotic additive and incubated at 37 °C in a humidified atmosphere at 5% CO2. The serous ovarian cancer cell line OV-90 was purchased at ATCC and grown in a 1:1 mixture of MCDB 105 medium containing a final concentration of 1.5 g/L sodium bicarbonate and Medium 199 containing a final concentration of 2.2 g/L sodium bicarbonate supplemented with 15% (v/v) fetal bovine serum and 1% (v/v) antibiotic-antimycotic additive and incubated at 37 °C in a humidified atmosphere at 5% CO2. All cell culture components were purchased from Gibco (LifeTechnologies).

### siRNA and transfections

MRE11 knockdowns were performed simultaneously with a pool of three different siRNA sequences (Microsynth, Switzerland) using RNAimax (Gibco by LifeTechnologies) according to the manufacturer’s instruction, each performed as two independent experiments. The target sequences (sense) were: GCUAAUGACUCUGAUGAUATT, GAGCAUAACUCCAUAAGUATT and GAUGCCAUUGAGGAAUUAGTT [24]. The controls were transfected with siRNA against luciferase (sense): CGUACGCGGAAUACUUCGATT. Cells were seeded 24 h after siRNA transfection and the treatment with the PARP inhibitor BMN673 was initiated after 48 h [23].

### Colony formation assays

The PARP inhibitor BMN673 was a gift from Biomarin Pharmaceuticals, USA. Survival assays were carried out as previously described [33, 34]. In brief, the previously transfected cells were seeded in six-well plates in triplicates at a concentration of 1000 cells per well. The PARP inhibitor treatment was started after 24 h and cells were continuously exposed to the PARP inhibitor (10–11 to 10–6 M) and the medium was replaced every 3 to 5 days. The controls were treated with the vehicle substance (DMSO). Cells cultures were grown until the controls reached 80–90% confluence after 10 to 15 days and then fixated with TCA 10%. After fixation, cell cultures were stained with sulforhodamine B (SRB) (Sigma) and a colorimetric assay was performed as described previously [33].

### Mutation analysis

The EOC cohort was previously characterized for *KRAS* and *TP53* gene mutations [35]. We analyzed *KRAS* exon 2 and 3 and *TP53* exon 5–8 by pyrosequencing using the GS Junior 454 platform.

### Immunoblotting

Protein extraction was performed with a SDS lysis buffer. The primary antibodies used for western blotting against MRE11 (D151 (sheep), gift from Steve Jackson, Cambridge) and beta-actin (mouse monoclonal antibody, Sigma). The incubation was followed by an HRP-conjugated secondary antibody (anti-sheep HRP from Santa-Cruz and anti-mouse HRP from Sigma), then followed by chemiluminescent detection (FemtoGlow Western, Michigan Diagnostics, Cat # FWPS02-500). Images were acquired electronically using a Fusion FX® (Vilber Lourmat, Marne-la-Vallée, France) detecting system.

### Statistical analysis

The statistical evaluation was performed with the SPSS software Version 21.0 (SPSS Inc., Chicago, IL, USA). The scoring data of MRE11, RAD50 and NBS1 were dichotomized into “negative” (no detection) and “positive” (weak to strong detection). The statistical significance of the association between these markers, the mismatch repair protein detection as well as clinicopathological parameters was assessed by Chi^2^ test and Fisher’s exact test. In addition, Spearman’s correlation was used to evaluate an association between MRE11, RAD50, NBS1 and mismatch repair proteins. The probability of overall survival as a function of time was determined by the Kaplan-Meier method. Differences in survival curves were compared by the log rank test. Sensitivity curves were calculated in GraphPad Prism Version 6 (GraphPad Software Inc., La Jolla, USA) and statistical differences between IC50 values were assessed using F-tests. The plotted values represent the mean surviving fraction and the error bars represent the standard error of mean (SEM) for two independent knockdown experiments, with each experiment perfomed in triplicates. *P*-values < 0.05 were considered as significant.

## Results

### MRN complex detection and clinicopathological parameters

**Fig. 1:**
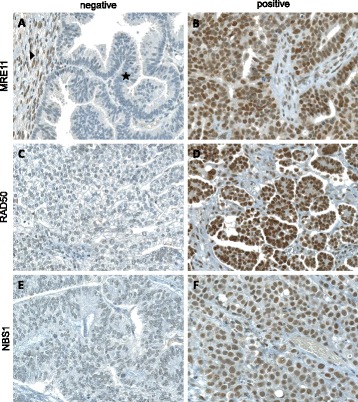
Immunohistochemical staining of MRE11 **a** & **b**, RAD50 (**c** & **d**) and NBS1 (**e** & **f**) (20× magnification). **a** Only complete absence of nuclear staining for MRE11 in tumor cells (⋆) was considered as negative staining. Adjacent normal tissue served as positive internal control (▶). Examples of undetectable RAD50 (**c**) and NBS1 (**e**) in tumor cells. Any nuclear staining of MRE11 (**b**), RAD50 (**d**) or NBS1 (**f**) was considered as positive

Immunohistochemical analysis of MRE11, RAD50 and NBS1 was successful in a maximum of 134 carcinomas. Staining results for some TMA spots were not obtained due to loss of tumor tissue during technical processing. MRE11, RAD50 and NBS1 immunohistochemical reactions showed only nuclear staining. The immunohistochemical data are summarized in Table 2. Representative images are shown in Fig. 1. Non-neoplastic stromal cells showed sustained staining of all three markers. For further statistical analysis, only completely absent staining (score 0) was considered as negative and any detectable staining (weak to strong) was considered positive.

**Table 2:** Immunohistochemical detection of MRN complex proteins

Immunohistochemical detection	*n* (%)
MRE11 (*n =* 136)	
Negative	49 (36.0)
Positive	87 (64.0)
RAD50 (*n =* 136)	
Negative	14 (10.3)
Positive	122 (89.7)
NBS1 (*n =* 135)	
Negative	45 (33.3)
Positive	90 (66.7)
MRN complex (*n =* 134)	
Negative	55 (41.0)
Positive	79 (59.0)

Statistically, we found a significant positive association between MRE11 and RAD50 as well as NBS1 detection (Table 3, *p <* 0.0001, Fisher’s Exact test). Further, a significant positive association was found between RAD50 and NBS1 (*p <* 0.0001, Fisher’s Exact test). Due to this association, we defined a combined loss of MRN complex proteins as loss of either of the proteins MRE11, RAD50 or NBS1.

**Table 3:** Association of the MRN complex proteins

	MRE11 negative n (%)	MRE11 positive n (%)	*p*-value
RAD50 (*n =* 135)			
Negative	14 (10.4)	0 (0)	
Positive	35 (25.9)	86 (63.7)	<0.001^a^
NBS1 (*n =* 135)			
Negative	39 (28.9)	6 (4.4)	
Positive	10 (7.4)	80 (59.3)	<0.001^a^
MMR (*n =* 133)			
Negative	25 (18.8)	3 (2.3)	
Positive	22 (16.5)	83 (62.4)	<0.001^a^

^a^Fisher’s Exact test

We evaluated the association of the MRN complex (combined detection of all three proteins) with clinicopathological factors. Lack of MRN complex detection was significantly associated with histological subtype and differentiation grade. According to the Kurman progression pathway model [32], lack of MRN protein detection occurred significantly more frequently in type I tumors (*p <* 0.001, Table 4).

**Table 4:** Association of MRN complex with clinicopathological factors and MMR status

Variable	*n*	MRN complex negative *n* (%)	*p*-value
FIGO stage			
Early (I & II)	40	20 (50)	0.017^a^
Late (III & IV)	88	32 (36.4)	
Histologic subtype			
High-grade serous	67	17 (25.4)	0.001^b^
Mucinous	14	9 (64.3)	
Clear cell	25	16 (64.0)	
Endometrioid	28	13 (46.4)	
Grade			
Low-grade	33	19 (57.6)	0.04^a^
High-grade	101	36 (35.6)	
Kurman model’			
Type I	58	35 (60.3)	0.0001^a^
Type II	76	20 (26.3)	
MMR status			
Negative	28	25 (89.3)	<0.0001^a^
Positive	103	27 (26.2)	
KRAS status			
wt	114	43 (37.7)	0.082^a^
Mutation	14	9 (64.3)	
TP53 status			
wt	58	24 (41.4)	0.9^a^
Mutation	70	28 (40.0)	

^**a**^Fisher’s Exact test; ^**b**^chi-square Pearson

### MRE11, RAD50 and NBS1 Detection and Mismatch Repair Protein Detection

We investigated mismatch repair protein expression by immunohistochemistry in a maximum of 133 ovarian carcinomas. Any lack of detection of one of the proteins MSH2 or MLH1 was integrated in a MMR deficiency state. MMR deficiency was observed in 21% of all EOC. All three MSH2 negative cases were also negative for MLH1.

MRE11, RAD50 and NBS1 were significantly associated with mismatch repair protein detection (*p <* 0.05, Fisher’s Exact test). The correlation among all proteins was confirmed in a bivariate (nonparametric) analysis (MRE11 and RAD50 (correlation coefficient 0.451; *n =* 135; *p*-value <0.001); MRE11 and NBS1 (correlation coefficient 0.741; *n =* 135; *p*-value <0.001); RAD50 and NBS1 (correlation coefficient 0.377; *n =* 134; *p*-value <0.001)), calculated with Spearman’s rho.

### MRN complex overall survival and response to chemotherapy

In univariate Kaplan-Meier analysis, FIGO stage, histologic subtype, grading and patient age were significant prognostic factors for overall survival (log rank, *p*-value <0.05 for each parameter). Detection of MRE11 (log rank, *p =* 0.28), RAD50 (log rank, *p =* 0.32), NBS1 (log rank, *p =* 0.9), MMR status (log rank, *p =* 0.79) was not associated with overall survival. NBS1 detection tended to be associated with a platinum-sensitive status (*p =* 0.058, Fisher’s Exact Test). MRE11 and RAD50 did not show any association with response to chemotherapy.

### Association of the MRN complex with KRAS and TP53 mutation status

Since *KRAS* and *TP53* gene mutations are important in ovarian carcinogenesis, we evaluated whether an association can be found with mutation status, which was recently described in our cohort [35]. Ovarian carcinomas with *KRAS* mutation showed frequent lack of NBS1 detection (*p =* 0.013, Fisher’s exact test) and a trend for MRE11 negativity (*p =* 0.08, Fisher’s exact test). No association with *TP53* mutation status was observed (Table 4).

### Knockdown of MRE11 sensitizes OCCC cells towards the PARP inhibitor BMN673 in vitro

**Fig. 2:**
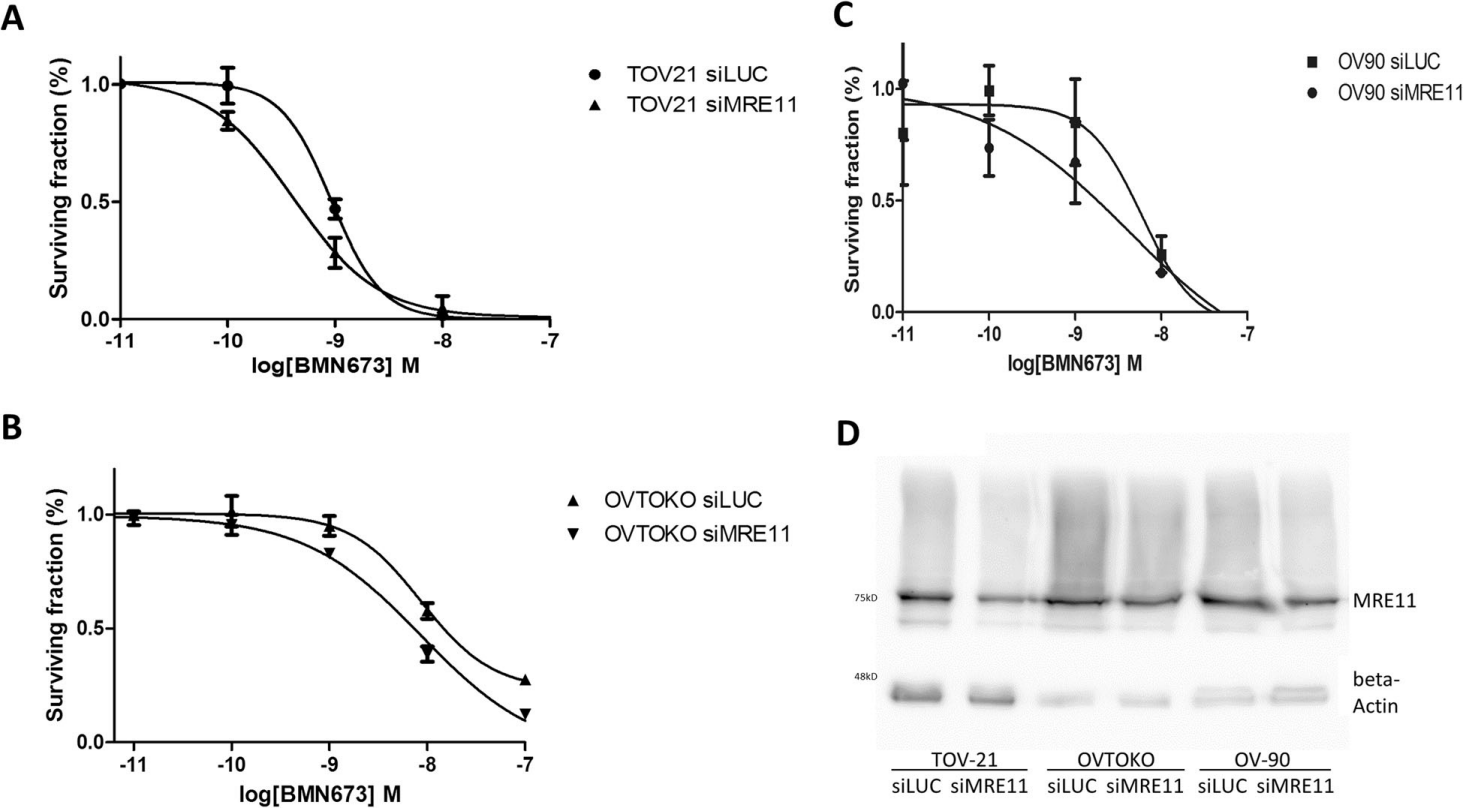
Sensitivity of the OCCC cell lines TOV-21, OVTOKO and ovarian serous cancer cell line OV-90 towards the PARP inhibitor BMN673 after knockdown of MRE11. Treatment with the PARP inhibitor BMN673 led to moderately decreased cell viability in (**a**) MRE11-depleted TOV21 (*p =* 0.0005) but not in (**b**) MRE11-depleted OVTOKO (*p =* 0.2) and OV-90 (**c**) (*p =* 0.8) compared to the respective controls. *P*-values indicate the differences in the IC50 values as calculated with an F-test using GraphPad Prism Version 6. The plotted values represent the mean surviving fraction and the error bars represent the standard error of mean (SEM). Western blot analysis (**d**) showed a decrease but not a loss of MRE11 expression in all the three cell lines after siRNA treatment and all the knockdown experiments were performed two times independently each. All the experiments were performed in triplicate 6-well plates

We assessed PARP inhibitor sensitivity in the MRE11-depleted cells by colony formation in triplicates. The efficiency of the siRNA knockdowns was verified by western blots as described before [21]. Upon depletion of MRE11, we observed a moderate decrease in cell viability in the presence of the PARP inhibitor BMN673 (Fig. 2). The IC50 values for BMN673 were 4.1 e–10 M in the MRE11-depleted TOV21 and 9.3 e–10 M in the controls (*p =* 0.0005). In OVTOKO IC50 was 5.4 e–9 M in the MRE11-depleted cell line and 7.5 e–9 M in the controls (*p =* 0.2).

## Discussion

In this study, immunohistochemical analysis showed a frequent lack of detection of the MRN complex (MRE11, RAD50, NBS1) in EOC. With our immunohistochemical approach, we provide evidence for lack of protein detection of RAD50 in 10% (14/136), NBS1 in 33% (45/135) and MRE11 in 36% (49/136) of EOC. In a combined MRN score, 44% (55/134) of all EOC have at least one undetectable protein of the three MRN proteins. Our finding is consistent with results of a previous study that demonstrated a reduced detection of MRE11, RAD50, and NBS1 in cancer tissue by immunohistochemistry as compared to the control group of healthy ovaries and serous cystadenomas [36]. Recently, we investigated these proteins in endometrial cancer (EC) and observed a lack of detection in up to 30% of the cases [9].

There are different mechanisms of MRN protein deficiency. Mutations of genes of the MRN complex have been described in other cancers, including colorectal and endometrial cancer. For MRE11, mutations of the intronic poly (T) sequence between exons 4 and 5 are frequent events in MSI-positive colorectal and endometrial cancers [37, 38]. However, in a recent next-generation sequencing study by exome-wide analysis in ovarian cancer, the prevalence of MRN gene mutations is uncommon. MRE11 accounted for only 3% of somatic mutations of HR genes and NBS1 only for 1% of germline mutations, respectively [6]. Therefore, different mechanisms which lead to undetectable MRN proteins might be suggested, such as epigenetic silencing [39] and post-transcriptional regulation by miRNA [40].

We observed an association between lack of MRN detection and MMR deficiency, assessed by MLH1 and MSH2 immunohistochemistry. MLH1 functions as a nuclear-encoded protein in mitochondrial mismatch repair, MSH2 functions in nuclear mismatch repair. Germline mutations in MSH2 and MLH1 account for the majority of HNPCC families implicating mismatch repair as etiology [41]. We have previously shown that nuclear immunoreactivity for MLH1, MSH2 or MSH6 proteins is retained in all microsatellite stable ovarian carcinomas [22]. 65% of microsatellite instable ovarian carcinomas displayed some detectable MLH1, MSH2 and MSH6, but a complete lack of MLH1, MSH2 or MSH6 detection was only seen in carcinomas with microsatellite instability [22, 42]. Therefore, our data indicate that lack of MRN detection can be associated with MMR deficiency in EOC. This is consistent with recent studies, showing that somatic mutations in *MRE11* are frequently detected in MSI-positive colorectal and endometrial cancers [6, 37, 38, 43]. Previously, we have observed that lack of MRN protein detection was also associated with undetectable MMR proteins in endometrial cancer [24]. This suggests that the components of the MRN complex might be a common target of MSI and might contribute to tumor progression.

In vitro experiments demonstrate that deficiency in HR by mutations in the MRE11-RAD50-NBS1 (MRN) complex may sensitize cancer cells to treatment with PARP inhibitors [26, 29, 44, 45]. We observed an increased sensitivity towards the PARP inhibitor BMN673 in the OCCC cell line TOV21 after knockdown of MRE11. A similar tendency was observed in the second OCCC cell line OVTOKO. The OCCC cell lines were chosen because of the higher rate of undetectable MRN proteins among the Type I ovarian cancer group in our TMA. The increase in PARP inhibitor sensitivity after MRE11 knockdown is in line with previous observations made in endometrial cancer cell lines [24]. However, the effect was less pronounced in the OCCC cell lines which may be caused by factors like reduced transfection efficiency in these cell lines.

Our data suggest that loss of MRE11 in ovarian and endometrial cancer may predict sensitivity to PARP inhibitor in vitro and supports further investigation on MRE11 as a predictive biomarker for PARP inhibitor treatment [24]. However, the deficiency of several proteins other than BRCA1/2 or the MRN complex involved in HR, such as RAD51, RAD54, DSS1, RPA1, ATR, ATM, CHK1, CHK2, FANCD2, FANCA or FANCC might also be sensitive to treatment with PARP inhibitors [6, 46].

In this study, MRN complex detection was not associated with response to chemotherapy or overall survival, which is consistent with previous studies in EOC [36] or endometrial cancer [24]. Although, loss of MRN complex is associated with impaired HR DNA repair [47], which renders cancer cells exquisitely sensitive to platinum-salts. This was recently shown in a large series of 390 ovarian cancer samples, where defects in HR were predictive for overall survival and primary platinum sensitivity [6]. The impact of MMR deficiency on chemotherapy resistance in EOC is controversial and investigated to a lesser extent [23]. The complex role and interaction of the MRN complex and MMR status has to be further evaluated.

Our data suggest that lack of MRN complex detection is more frequent in nonserous histologies (type I EOC) than in high-grade serous EOC [32]. Undetectable MRN protein was only weakly associated with low differentiation grade (*p <* 0.05). We classified the histological grade as low-grade (including well to moderately differentiated endometrioid and mucinous carcinomas) and high-grade (combining high-grade serous, clear cell and poorly differentiated endometrioid cancer). Importantly, undetectable MRN complex showed stronger association with the Kurman model of two progression pathways: Type I low-grade ovarian carcinomas (low-grade serous cancer, mucinous, endometrioid and clear cell cancer), which are characterized by mutations in genes like *KRAS*, *BRAF*, *ERBB2*, *PTEN*, *PI3KCA*, *CTNNB1*, *ARID1A* and *PPP2R1A*, in the absence of *TP53* mutations. These type I EOC more often showed an undetectable MRN complex than the aggressive type II high-grade ovarian carcinomas (especially high-grade serous cancer) with a high frequency of *TP53* mutations [32]. Type II EOC typically occurs in patients with germline *BRCA1* and *BRCA2* mutations, which are treated with PARP inhibitors. Our data suggest that patients with type I EOC may also benefit from PARP inhibitors.

Finally, we searched for an association between gene mutations of *TP53* and *KRAS* and the protein detection of the MRN complex. Lack of NBS1 protein detection was associated with *KRAS* mutation, presumably due to the strong association between type I EOC and lack of MRN protein detection. In a previous study, a relation of NBS1 to the RAS/RAF/MER/ERK cascade and its influence in cell proliferation was shown [34]. Thus, NBS1 is not only involved in DNA repair but also in other cellular processes such as signalling and proliferation. It was further reported that p53 mutants interact with the MRN complex [48]. However, in our cohort we did not observe an association between *TP53* mutated ovarian carcinomas and MRN complex detection. This can be explained by the weak association of MRN deficiency with type II EOC.

## Conclusion

In conclusion, lack of protein detection of the MRN complex occurs more frequently in low-grade EOC and is associated with MMR deficiency. A potential role of MRN as a biomarker for therapies targeting cancers with BRCAness such as PARP inhibitors needs to be examined in clinical trials.
